# Video laryngoscopy improves intubation success and reduces esophageal intubations compared to direct laryngoscopy in the medical intensive care unit

**DOI:** 10.1186/cc13061

**Published:** 2013-10-14

**Authors:** Jarrod M Mosier, Sage P Whitmore, John W Bloom, Linda S Snyder, Lisa A Graham, Gordon E Carr, John C Sakles

**Affiliations:** 1 Department of Emergency Medicine, University of Arizona, Tucson, AZ, USA; 2 Department of Medicine, Section of Pulmonary, Critical Care, Allergy and Sleep, University of Arizona, 1609 N Warren, FOB 122C, Tucson, AZ 85719, USA

## Abstract

**Introduction:**

Tracheal intubation in the Intensive Care Unit (ICU) can be challenging as patients often have anatomic and physiologic characteristics that make intubation particularly difficult. Video laryngoscopy (VL) has been shown to improve first attempt success compared to direct laryngoscopy (DL) in many clinical settings and may be an option for ICU intubations.

**Methods:**

All intubations performed in this academic medical ICU during a 13-month period were entered into a prospectively collected quality control database. After each intubation, the operator completed a standardized form evaluating multiple aspects of the intubation including: patient demographics, difficult airway characteristics (DACs), method and device(s) used, medications used, outcomes and complications of each attempt. Primary outcome was first attempt success. Secondary outcomes were grade of laryngoscopic view, ultimate success, esophageal intubations, and desaturation. Multivariate logistic regression was performed for first attempt and ultimate success.

**Results:**

Over the 13-month study period (January 2012-February 2013), a total of 234 patients were intubated using VL and 56 patients were intubated with DL. First attempt success for VL was 184/234 (78.6%; 95% CI 72.8 to 83.7) while DL was 34/56 patients (60.7%; 95% CI 46.8 to 73.5). Ultimate success for VL was 230/234 (98.3%; 95% CI 95.1 to 99.3) while DL was 52/56 patients (91.2%; 95% CI 81.3 to 97.2). In the multivariate regression model, VL was predictive of first attempt success with an odds ratio of 7.67 (95% CI 3.18 to 18.45). VL was predictive of ultimate success with an odds ratio of 15.77 (95% CI 1.92 to 129). Cormack-Lehane I or II view occurred 199/234 times (85.8%; 95% CI 79.5 to 89.1) and a median POGO (Percentage of Glottic Opening) of 82% (IQR 60 to 100) with VL, while Cormack-Lehane I or II view occurred 34/56 times (61.8%; 95% CI 45.7 to 71.9) and a median POGO of 45% (IQR 0 to 78%) with DL. VL reduced the esophageal intubation rate from 12.5% with DL to 1.3% (*P* = 0.001) but there was no difference in desaturation rates.

**Conclusions:**

In the medical ICU, video laryngoscopy resulted in higher first attempt and ultimate intubation success rates and improved grade of laryngoscopic view while reducing the esophageal intubation rate compared to direct laryngoscopy.

## Introduction

Tracheal intubation is a lifesaving procedure frequently performed in the intensive care unit (ICU). Multiple anatomic and physiologic factors make airway management in the ICU more challenging than elective intubation in the controlled setting of the operating room [1-3]. Patients are often hemodynamically unstable or hypoxemic, and may have anatomic characteristics, such as distorted airway anatomy or abnormal body habitus, associated with difficult intubation [3-5]. Traditionally, orotracheal intubation has been performed in the ICU with a direct laryngoscope (DL), which requires alignment of the oral, pharyngeal and laryngeal axes to allow direct visualization of the glottic inlet. When performed in the ICU, DL has been associated with a high incidence of difficult intubations and complications [6-10]. Video laryngoscopy (VL) was developed to obviate the need for direct visualization of the glottic inlet by transporting the view of the airway to a monitor via a micro video camera placed on the under surface of the blade. This allows the operator to see “around the corner” when alignment of the axes is difficult. VL has been shown to improve first attempt success and grade of laryngoscopic view when compared to DL in the simulation lab, operating room, emergency department and ICU [11-22]. When used by anesthesiologists in the ICU setting, VL improved success rate and grade of view compared to DL in the presence of difficult airway predictors [22].

Our study compares the difference in success rates and glottic visualization between direct laryngoscopy and video laryngoscopy using either the GlideScope (GVL) (Verathon Medical, Bothell, WA, USA) or C-MAC (CMAC) (Karl Storz, Tuttlingen, Germany) for intubations performed by non-anesthesiologists in an academic medical ICU setting.

## Materials and methods

### Study design

This analysis included 317 consecutive ICU intubations prospectively recorded in a continuous quality improvement (CQI) database from 1 January 2012 to 31 January 2013. This project was granted exemption of consent and approved by the University of Arizona Institutional Review Board.

### Study setting

This study was conducted at a major academic referral center with a 20+ bed medical ICU, which is staffed by two teaching teams. This ICU service is affiliated with Accreditation Council for Graduate Medical Education (ACGME) accredited three-year pulmonary/critical care medicine (Pulm/CCM) and two-year critical care medicine fellowship programs with a total of 14 fellows.

### Selection of participants

#### Patients

The GVL was introduced into our ICU prior to the start of the CQI database and is the primary video laryngoscope located on multiple ICU wards where medical ICU patients may overflow. The CMAC was introduced in our ICU in March 2012 and video laryngoscopy has become the preferential device for intubations in the ICU over this time period. Only patients who were intubated using the GVL (size 3 or 4), CMAC (size 3 or 4) or the Macintosh DL (size 3 or 4) as the initial device from 1 January 2012 until 31 January 2013 were included in this study. Intubations utilizing the Miller blade, bronchoscope or other video laryngoscopes were excluded from analysis.

#### Operators

Each teaching team is staffed with an attending (Pulm/CCM or CCM), a fellow (Pulm/CCM post-graduate year (PGY) 4, 5 or 6 or CCM PGY 4 or 5), and residents (Internal Medicine PGY 1 and 3, Emergency Medicine PGY 2, and occasionally Family Medicine PGY 2). The majority of intubations are performed by residents, Pulm/CCM or CCM fellows under attending intensivist supervision. All fellows in this program participate in an ongoing didactic airway curriculum consisting of lectures during dedicated conference time discussing airway management algorithms, devices and cases. A simulation laboratory is also available to all fellows to practice using a variety of airway devices.

### Methods of measurement

Following each intubation, the operator completed a data collection form, which included the following information: patient demographics, operator specialty, operator PGY, indication for intubation, method of intubation, paralytic agent, sedative agent, device(s) used, presence of certain difficult airway characteristics (DACs), pre-oxygenation methods, the Cormack-Lehane (CL) view and Percentage of Glottic Opening (POGO) score of the airway, number of attempts at intubation and the outcome of each attempt, including complications.

Methods of intubation included rapid sequence intubation (RSI) in which a paralytic agent was used, oral intubation in which a sedative agent only was used (sedation only (SED)), and oral intubation in which no medications were used (oral intubation without sedation (OTI)).

Standard pre-operative difficult airway predictors, such as the Mallampati score, thyromental distance and neck mobility, have been shown to be challenging to apply in the emergency setting due to lack of patient cooperation and the urgency to complete the intubation rapidly [23,24]. Thus, we developed a list of DACs that were feasible for the operator to determine prior to intubation in an emergent setting by simple examination of the patient. These include both anatomic and physiologic difficult airway characteristics. The anatomic DACs include: the presence of blood, vomit or secretions in the airway, cervical immobility (intrinsic or due to a cervical collar), obesity, large tongue, short neck, small mandible, facial or neck trauma, airway edema and limited mouth opening. Physiologic DACs include hemodynamic instability and hypoxemia, which may make the situation more challenging, rather than the procedure itself.

An intubation attempt was defined as insertion of the laryngoscope blade into the oropharynx regardless of whether an attempt was made to pass the endotracheal tube (ETT). Each attempt was documented with one of three possible outcomes: 1) successful tracheal intubation (no additional attempts required), 2) inability to intubate (additional attempt(s) required), or 3) inadvertent esophageal intubation (additional attempt(s) required). Successful intubation was defined as correct placement of the ETT in the trachea as confirmed by an end-tidal CO_2_ capnometry, pulse oximetry, chest auscultation, observation of chest excursion, absence of epigastric sounds and misting of the ETT. If there was uncertainty about ETT placement by the operator and the tube was removed and replaced, the attempt was considered to have been an esophageal intubation. First attempt success was defined as successful tracheal intubation, as defined above, on the initial attempt. Ultimate success was defined as successful tracheal intubation with the initial device, regardless of the number of attempts required.

When the CMAC was used as a direct laryngoscope, the attempt was considered a VL attempt regardless of whether the operator looked at the monitor during the attempt as the supervisor was able to provide real-time feedback as if it were performed by video laryngoscopy.

Complications included hypotension, desaturation, esophageal intubation, dental trauma, mainstem intubation, witnessed aspiration, cuff leak, pneumothorax or other. For the purposes of this analysis, complications evaluated included first attempt esophageal intubation, any attempt esophageal intubation, first attempt and any attempt desaturation.

All data forms were reviewed by the primary author. If the forms had any missing data, they were returned to the operator for completion. If information on the form contained inconsistencies, the operator was interviewed by the primary author for clarification.

The data were then entered into the electronic database (Excel for Macintosh 2011 (Microsoft, Redmond, WA, http://www.microsoftstore.com)) and ultimately transferred to Stata for analysis (Stata version 12; StataCorp, College Station, TX, USA)*.*

### Outcome measures

The primary outcome measured was successful first attempt intubation. Secondary outcome measures were ultimate successful intubation, initial laryngoscopic grade of view (Cormack-Lehane view, POGO score), and complications.

### Primary data analysis

Summary statistics were generated for patient, intubation and operator characteristics. Multivariate logistic regression analyses were performed for both first attempt success and ultimate successful intubation. The predictor variable of interest was intubation device (VL or DL). Other predictor variables added to each model to adjust for confounding included age, sex, method of intubation, presence of DACs, total DACs, operator specialty and operator PGY. Summary statistics and regression analyses were calculated using STATA 12 for Macintosh (StataCorp LP, College Station, TX, USA, http://www.stata.com). A 95% confidence interval for all counts and proportions was calculated using the “exact” method.

## Results

Over the 13-month study period, a total of 290 patients were intubated with an initial device of the GVL, CMAC or DL. Of these, 234 patients were intubated using a video laryngoscope (68 GVL, 166 CMAC), and 56 patients were intubated with DL. The patient, intubation and operator characteristics of the VL group and the DL group were very similar (see Table 1). The mean age, proportion of males to females and indication for intubation were similar between groups. However, significant differences existed in method of intubation, operator specialty and PGY level. Regarding method of intubation, the majority of patients were intubated by RSI and this did not differ between groups (VL 76.5%; DL 86.0%; *P* = 0.15; Table 1). More patients in the VL group were intubated using sedation only (VL 22.7% vs. DL 3.6%; *P* = 0.0005; Table 1).

**Table 1:** Patient and operator demographics

**Characteristic**	**VL %, (n = 234)**	**95% CI**	**DL %, (n = 56)**	**95% CI**	***P*-value**
**Mean Age, years**	59.5 (IQR 23 to 90 )	57.7 to 61.3	61.8 (IQR 40 to 82)	58.0 to 65.7	0.27*
**Gender**					0.46
Male	56.0% (131)	45.7 to 65.9	50.0% (28)	38.8 to 59.1	
**DACs**					
Total DACs (median)	2	IQR 1 to 3	1	IQR 1 to 2	0.18
None	21.0% (49)	16.1 to 26.9	21.4% (12)	10.9 to 32.8	0.52
Cervical immobilization	1.7% (4)	0.4 to 4.3	5.4% (3)	1.2 to 14.9	0.13
Blood in Airway	19.2% (45)	14.4 to 24.9	8.9% (5)	3.0 to 19.6	0.07
Vomit in Airway	6.0% (14)	3.3 to 9.8	7.1% (4)	2.0 to 17.3	0.76
Facial/neck Trauma	0.43% (1)	0.01 to 2.3	0% (0)	0 to 6.4	1.00
Obesity	29.5% (69)	23.7 to 35.8	17.86% (10)	8.9 to 30.4	0.10
Short Neck	28.2 % (66)	22.5 to 34.4	19.6% (11)	10.2 to 32.4	0.24
Large Tongue	16.2% (38)	11.8 to 21.6	14.3% (8)	6.4 to 26.2	0.84
Airway Edema	8.6% (20)	5.2 to 12.9	10.7% (6)	4.0 to 21.9	0.61
Small Mandible	16.2% (38)	11.8 to 21.6	28.6% (16)	17.3 to 42.2	0.05
Limited Mouth Opening	8.2% (19)	5.0 to 12.4	3.6% (2)	0.4 to 12.3	0.39
Secretions	11.5% (27)	7.4 to 15.9	3.6% (2)	0.4 to 12.3	0.04
Hemodynamic Instability	26.5% (62)	21.0 to 32.6	16.1% (9)	7.6 to 28.3	0.12
Hypoxemia	27.8% (65)	22.1 to 34.0	34.0% (19)	21.8 to 47.8	0.41
**Reason for Intubation**					0.42
Airway Protection	20.1% (47)	15.4 to 26.0	21.4% (12)	11.6 to 34.4	0.85
Respiratory Failure	65.0% (152)	58.5 to 71.1	60.7% (34)	46.8 to 73.5	0.64
Cardiac Arrest	1.7% (4)	0.5 to 4.3	7.1% (4)	2.0 to 17.3	0.05
Patient Control	1.3% (3)	0.3 to 3.7	1.8% (1)	0.05 to 9.6	0.58
Hypoxemia	9.0% (21)	5.6 to 13.4	7.1% (4)	2.0 to 17.3	0.80
Hemodynamic Instability	1.7% (4)	0.5 to 2.3	1.8% (1)	0.05 to 9.6	1.0
Severe Acidosis	1.3% (3)	0.3 to 3.7	0% (0)	0.0 to 6.0	1.0
**Method of Intubation**					<0.001
RSI	76.5% (179)	70.5 to 81.8	86.0% (48)	73.8 to 93.6	0.15
SED	22.7% (53)	17.5 to 28.6	3.6% (2)	0.4 to 12.3	0.0005
OTI	0.9% (2)	0.1 to 3.0	10.7% (6)	0.4 to 21.9	0.0008
**Operator Specialty**					<0.001
Internal Medicine	28.2% (66)	22.5 to 34.4	12.5% (7)	5.2 to 24.1	0.02
Emergency Medicine	13.7% (32)	9.5 to 18.8	35.7% (20)	23.4 to 49.6	0.0003
Pulmonary/Critical Care	51.7% (121)	45.1 to 58.3	32.1% (18)	20.3 to 46.0	0.01
Critical Care Medicine	6.4% (15)	3.6 to 10.4	19.6% (11)	10.2 to 32.4	0.006
**Operator PGY Level**					0.001
1	9.4% (22)	5.9 to 13.8	7.1% (4)	2.0 to 17.3	0.80
2	17.1% (40)	12.5 to 22.5	37.5% (21)	25.0 to 51.5	0.001
3	15.4% (36)	11.0 to 20.7	3.6% (2)	0.4 to 12.3	0.01
4	21.8% (51)	16.7 to 27.6	28.6% (16)	17.3 to 42.2	0.29
5	25.2% (59)	19.8 to 31.3	19.6% (11)	10.2 to 32.4	0.49
6	9.4% (22)	6.0 to 13.9	0.0% (0)	0.0 to 6.4	0.01
Attending	1.7% (4)	0.5 to 4.3	3.6% (2)	0.4 to 12.3	0.33

*T-test. DACs, difficult airway characteristics; DL, direct laryngoscope/laryngoscopy; OTI, oral intubation without sedation; PGY, post-graduate year; RSI, Rapid Sequence Intubation; SED, sedation only; VL, Video laryngoscope/laryngoscopy.

Only a minority of patients (21.0%) had none of the pre-determined DACs and there was no difference between groups (VL 21.0%; DL 20.4%; *P* = 0.52; Table 1). The median number of DACs per patient differed between groups but did not reach statistical significance. There were significant differences with respect to specific DACs. More patients in the DL group were identified with a small mandible as a DAC (28.6%, 95% CI 17.3 to 42.2) compared to the VL group (16.2%, 95% CI 11.8 to 21.6). However, the VL group had more patients with secretions (11.5%, 95% CI 7.4 to 15.9) than the DL group (3.6%, 95% CI 0.4 to 12.3). When an internal medicine resident performed the intubation, they used VL 90.4% (95% CI 81.2 to 96.1) of the time and DL 9.6% (95%CI 3.9 to 18.8) of the time. Emergency medicine residents used VL 61.5% (95% CI 47.0 to 74.7) of the time and DL 38.5% (95% CI 25.3 to 53.0). Pulmonary/critical care fellows and attendings used VL 87% (95% CI 80.3 to 93.1) and DL 13% (95% CI 7.9 to 19.7) of the time. Critical care fellows and attendings used VL 57.7% (95% CI 36.7 to 76.6) and DL 43.3% (95% CI 23.4 to 63.1) of the time.

The use a VL varied with level of training. When a PGY 1 performed the intubation, they used VL 84.6% (95% CI 65.1 to 95.6) of the time and DL 15.4% (95% CI 4.4 to 34.9). PGY 2 residents used VL 65.6% (95% CI 52.0 to 77.3) and DL 34.4% (95% CI 22.3 to 47.7), PGY 3 used VL 95% (95% CI 82.3 to 99.4) and DL 5% (95% CI 0.6 to 17.8), PGY 4 used VL 76% (95% CI 64.1 to 85.7) and DL 24% (95% CI 14.3 to 35.9), PGY 5 used VL 84% (95% CI 73.6 to 91.9) and DL 16% (95% CI 8.1 to 26.4), PGY 6 used VL 100% (95% CI 84.6 to 100.0), and attendings used VL 67% (95% CI 22.3 to 95.7) and DL 33% (95% CI 4.3 to 77.7) of the time.

Table 2 demonstrates success rates for VL and DL by number of attempts. Video laryngoscopy was successful on first attempt in 184 of 234 patients (78.6%; 95% CI 72.8 to 83.7) while direct laryngoscopy was successful in 34 of 56 patients (60.7%; 95% CI 46.8 to 73.5). Video laryngoscopy was ultimately successful in 230 of 234 patients (98.3%; 95% CI 95.1 to 99.3) while direct laryngoscopy was ultimately successful in 52 of 56 patients (91.2%; 95% CI 81.3 to 97.2). First attempt success rates for VL and DL by number of DACs are shown in Table 2. For patients with no DACs, there was no statistically significant difference between devices in regards to first attempt success rate (VL 84% (95% CI 76.7 to 86.9) vs. DL 75% (95% CI 73.0 to 92.8)). For patients with one or more DACs, VL demonstrated a higher first attempt success rate than DL (Table 2). Table 3 demonstrates first and ultimate success rates by resident and fellow/attending training levels.

**Table 2:** Success rate by number of difficult airway characteristics and number of attempts

**Number of DACs**	**VL %, (n = 234)**	**95% CI**	**DL %, (n = 56)**	**95% CI**	***P*-value**
0	84% (41)	76.7 to 86.9	75% (9)	73.0 to 92.8	0.67
1	80.0% (40)	76.9 to 87.0	55.6% (10)	45.7 to 65.9	0.04
2	83.3% (45)	75.3 to 85.7	46.2 (6)	36.9 to 57.2	0.009
2+	76.3% (103)	69.0 to 83.0	57.7% (15)	39.5 to 73.0	0.04
**Attempts**					
1	78.6% (184)	72.8 to 83.7	60.7% (34)	46.8 to 73.5	0.009
2	96.2% (225)	92.9 to 98.2	85.7% (48)	73.0 to 92.7	0.007
3	97.4% (228)	94.5 to 99.0	89.3% (50)	77.0 to 95.1	0.01
>3	98.3% (230)	95.1 to 99.3	91.2% (52)	81.3 to 97.2	0.04

Table 2: Compares VL to DL by number of DACs and number of attempts. As shown above, the success rate of DL falls sharply with each successive DAC, while the success rate of VL stays consistently higher. For patients requiring multiple attempts, VL demonstrated a higher success rate at each attempt. DACs, Difficult airway characteristics; DL, direct laryngoscope/laryngoscopy; VL, Video laryngoscope/laryngoscopy.

**Table 3:** First attempt and ultimate success rates by level of training

**Training level**	**First attempt success - DL**	**First attempt success - VL**	***P*-value**	**Ultimate success- DL**	**Ultimate success- VL**	***P*-value**
Residents (PGY 1 to 3)	59% (16/27)	73% (72/98)	0.16	93% (25/27)	97% (95/98)	0.29
Fellows/Attendings (PGY 4+)	62% (18/29)	82% (112/136)	0.02	93% (26/28)	99% (134/136)	0.03

Table 3: Compares the first attempt and ultimate success rates between VL and DL by residents and fellows/attendings. DL, direct laryngoscope/laryngoscopy; PGY, post-graduate year; VL, video laryngoscope/laryngoscopy.

In the multivariate regression model, VL was predictive of first attempt success with an odds ratio of 7.67 (95% CI 3.18 to 18.45). Male gender (OR 2.09; 95% CI 1.07 to 4.07) was a demographic variable that predicted first attempt success. Compared to non-intensivist specialties, intubation by pulmonary/critical care medicine and critical care medicine specialists predicted first attempt success (OR 5.59; 95% CI 1.09 to 28.64). Method of intubation, operator PGY, and DAPs were not statistically significant predictors of first attempt success in the model (Table 4). In the multivariate regression model of ultimate success, VL was predictive of ultimate success with an odds ratio of 15.77 (95% CI 1.92 to 129).

**Table 4:** Multivariate Regression Model for first attempt success

**Variable**	**OR**	**95% CI**	***P*-value**
Video laryngoscope as first device*	7.67	3.18 to 18.45	<0.001
Age	1.02	1.00 to 1.04	0.04
Gender**	2.09	1.07 to 4.07	0.02
**Method**			
No Medications	(Reference)		
RSI	0.72	0.08 to 6.00	0.76
Sedation only	0.24	0.02 to 2.42	0.22
**Operator Specialty**			
Non-intensivist	(Reference)		
Pulmonary and Critical Care Medicine	5.59	1.09 to 28.64	0.04
**Operator PGY**			
1	(Reference)		
2	3.19	0.97 to 10.51	0.06
3	0.66	0.20 to 2.13	0.48
4	0.53	0.06 to 4.33	0.55
5	0.36	0.04 to 3.22	0.36
6	0.51	0.05 to 4.88	0.56
Attending	3.0	0.11 to 77.74	0.51
**Difficult Airway Predictors**			
Total DACs	0.44	0.13 to 1.51	0.20
Blood	1.79	0.39 to 8.33	0.45
Vomit	0.84	0.15 to 4.66	0.84
Cervical Immobilization	6.79	0.36 to 125.46	0.20
Airway edema	0.60	0.11 to 3.25	0.55
Small mandible	1.05	0.22 to 5.03	0.95
Obesity	1.39	0.29 to 6.77	0.68
Large tongue	3.31	0.64 to 17.09	0.15
Short neck	1.63	0.36 to 7.25	0.52
Hemodynamic instability	1.58	0.37 to 6.46	0.52
Hypoxemia	2.78	0.65 to 11.85	0.16
Limited mouth opening	2.74	0.45 to 16.65	0.27
Secretions	1.46	0.27 to 7.7	0.65

In this multivariate regression model controlling for the confounding variables, the odds ratio of first attempt success using VL was 7.67, compared to DL. See text for explanation.

*Reference = DL. **Reference = Male. DACs, Difficult airway characteristics; PGY, post-graduate year.

When using VL, operators obtained a Cormack-Lehane I or II view 199 of 234 times (85.8%; 95% CI 79.5 to 89.1) and a median POGO of 82% (IQR 60 to 100). DL obtained a Cormack-Lehane I or II view 34 of 56 times (61.8%; 95% CI 45.7 to 71.9) and a median POGO of 45% (IQR 0 to 78%) (Table 5).

**Table 5:** Grade of laryngoscopic view

**Grade of view**	**VL %, (n = 234)**	**95% CI**	**DL %, (n = 56)**	**95% CI**	***P*-value**
CL I to II	85.8% (199)	79.5 to 89.1	61.8% (34)	45.7 to 71.9	<0.001
POGO (median, IQR)	82%	62% to 100%	45%	0 to 78%	0.0001

Table 5: Demonstrates improved laryngoscopic view with VL compared to DL. CL, Cormack-Lehane; DL, direct laryngoscope/laryngoscopy; POGO, percentage of glottis opening; VL, Video laryngoscope/laryngoscopy.

DL was responsible for five (8.9%) first attempt esophageal intubations while VL was responsible for three (1.3%) first attempt esophageal intubations (*P* = 0.008). For patients with multiple attempts with DL, there were 7 (12.5%) esophageal intubations while there were no other esophageal intubations with VL (*P* = 0.001). There was no significant difference in first attempt (DL 18%, VL 23%) or any attempt desaturation (DL 35%, VL 44%) between DL and VL.

## Discussion

In this study of intubations performed in the ICU by mostly trainees with limited experience, video laryngoscopy demonstrated significantly improved first attempt and ultimate success rates, grade of laryngoscopic view, and decreased esophageal intubations compared to direct laryngoscopy. These differences were despite the majority of DL intubations being performed by the relatively more skilled operators (EM residents and critical care medicine fellows/attendings) and more patients in the DL category receiving RSI, which improves overall intubating conditions and grade of laryngoscopic view.

Airway management in the ICU can be very risky due to difficult anatomic features of the patient and decompensated cardiopulmonary physiology. Several large series have demonstrated significant complications, including hypoxemia, esophageal intubation, aspiration, hypotension, dysrhythmias and cardiac arrest, in patients undergoing tracheal intubation in the ICU. Hypoxemia was found to occur in 19 to 26% of cases, cardiac arrest in 2%, and overall complication rates seen in 28 to 39% of cases [4,6,8,10,25,26]. Furthermore, the likelihood of encountering a difficult airway due to poor glottic exposure in this population was as high as 20% in one recent series [22]. In light of the difficulty encountered in this population, first attempt success during tracheal intubation is of critical importance as adverse events, including neurologic insult, severe hypoxemia, cardiac arrest, and death, increase with each successive attempt [9,27-29].

Improving first-attempt success and ultimately reducing the risk of complications during ICU intubations presents a unique challenge. Intubation in the ICU can be both technically and circumstantially difficult. Investigators have attempted to identify the incidence, risk, and prevention of hemodynamic instability and hypoxemia [30-32] as, despite pre-intubation optimization, they have been shown to lead to increased odds of complications [33]. Checklists and prediction scores developed to improve the circumstantial difficulty of airway management in the ICU have had only modest outcomes [7,34].

The procedural difficulty of intubation is multifactorial. ICU patients often possess anatomic and physiologic factors that can interfere with alignment of the oral, pharyngeal and laryngeal axes or impair direct visualization of the airway using a direct laryngoscope. The level of experience and training of the operator has been identified as a potential indicator of a more difficult intubation [3,5,6,8-10,34,35]. However, difficult intubation rates and complication rates remain high even in the presence of experienced anesthesiologists, with no significant difference in complication rates between anesthesiologists and non-anesthesiologists [3,6,8]. This raises the question as to whether the device used to make the attempt, rather than the training of the operator, can close the gap between experienced anesthesiologists and non-anesthesiologists to improve first attempt success rates.

While data comparing VL and DL in the Emergency Department and Operating Room [18,20,21,36-44] have shown improved performance with video laryngoscopy, data in the ICU are limited. Noppens *et al*. published the first ICU comparison of DL to the C-MAC in terms of first attempt success rates, glottic exposure, and complications during 274 intubations in a surgical ICU population [22]. The operators were described as junior level, senior level and attending level anesthesia-trained physicians; all intubations included in the study were rapid sequence intubations. Overall, there was no statistically significant difference in first attempt success rates between DL and C-MAC (80% vs. 88%); however, in patients with at least one difficult airway predictor, such as obesity, short neck, small mouth, large tongue or cervical immobility, the C-MAC substantially improved first attempt success rates (56% vs. 79%) as well as glottic exposure. There were no differences in overall complication rates or rates of hypoxemia between groups. Two recent studies from the same institution compared VL intubations with historical control cohorts intubated with DL and demonstrated a reduction in airway related complications when pulmonary and critical care fellows intubated with GVL [45,46].

Our data extend the existing literature comparing VL to DL to the ICU setting. In this study, we demonstrate a greatly improved first attempt success rate and glottic view when a video laryngoscope was used. This improved success rate and decrease in esophageal intubation rate was despite relatively inexperienced providers using mostly video laryngoscopy. Interestingly, the success rate of VL remained high in the presence of increased difficult airway predictors while the success rate of DL declined. Lastly, the first attempt success rate despite level of experience and training approaches that demonstrated by Noppens, suggesting that video laryngoscopy may be crucial for improving the airway management experience in the ICU settings, despite who is performing the procedure.

In addition to the apparent benefit of VL in the presence of difficult airway predictors, success rates are also improved when VL is utilized by relatively inexperienced operators. A prospective, randomized trial comparing intubation of elective operative patients with GVL versus DL in the hands of interns, nurses, new paramedics and medical students, with only cursory manikin training beforehand, showed marked improvement in overall success rates with GVL (93% vs. 51%) [47]. In their meta-analysis of over 1998 patients, Griesdale and colleagues showed that while there is no difference in first attempt or overall success comparing DL to VL among expert users (senior anesthesia residents or anesthesia attendings, for example), non-experts (including house staff, medics, nurses, and medical students) are afforded quicker intubation times and a higher first attempt success rate using VL [48].

The results of this study demonstrate improved first attempt success rates, improved overall success rates, and improved glottic visualization using VL compared to DL in an ICU population when operated by non-anesthesiology intensivists. The results of this study are important for two major reasons. First, these data further strengthen the body of literature supporting the use of video laryngoscopy as the primary device of choice when intubating critically ill patients, especially those with difficult airway characteristics. In keeping with the previous literature our first attempt success rate with VL was 79%, and in our study the use of VL was nearly eight times more likely to result in a successful intubation on the first attempt. In the presence of one or more difficult airway characteristics, VL improved while DL declined. Second, compared to anesthesia providers in an ICU setting, non-anesthesiology providers can achieve similar rates of first attempt and overall success using VL in the ICU.

There are several important limitations to this study. As described in Table 1, patients in the DL and VL groups differed significantly in terms of method of intubation (that is, rapid sequence versus sedation only or no sedation), operator training level and operator specialty, and certain difficult airway characteristics, all of which may have impacted the success rates within each group. As this was an observational study, there may have been significant selection bias in terms of which patients underwent intubation with which device given each operator’s training level, specialty and preferences. With multivariate regression, we controlled for multiple different airway predictors, but there are other factors involved in selecting intubation method and device in a non-randomized fashion. Finally, it has been suggested that nearly 60 DL attempts are needed for a trainee to acquire a reliable level of performance with this technique [49]; less familiarity and experience with DL among our operators likely accounts for its relatively low success rate in our study compared to prior studies [22,36,42,44]. Conversely, particular strengths of our study include a relatively large number of patients and real-world applicability given the variation in patients, methods, devices and operators.

## Conclusion

This analysis of medical ICU intubations demonstrates improved first attempt success, overall success, and glottic visualization using VL compared to DL when performed by non-anesthesiologists. Randomized trials to confirm these results are warranted.

## Key messages

● Intubations in the ICU can be risky due to complex anatomic and physiologic factors.

● Direct laryngoscopy requires direct visualization of the glottic inlet to visualize correct placement of the endotracheal tube, which can be particularly difficult in this patient population.

● Video laryngoscopy provides an indirect view of the glottic inlet, which allows the operator to visualize the airway despite complex difficult airway characteristics.

● When used by non-anesthesiologists, video laryngoscopy in this study narrows the gap in success rate and esophageal intubations between anesthesiologists and non-anesthesiologists reported previously in the literature.

## Abbreviations

ACGME: Accreditation Council for Graduate Medical Education; CCM: Critical Care Medicine; CL: Cormack-Lehane; CMAC: C-MAC Video laryngoscope; CQI: Continuous quality improvement; DACs: Difficult airway characteristics; DL: Direct laryngoscope/laryngoscopy; ETT: Endotracheal tube; GVL: GlideScope; ICU: Intensive care unit; OTI: Oral intubation without sedation; PGY: Post graduate year; POGO: Percentage of glottic opening; Pulm/CCM: Pulmonary and Critical Care Medicine; QI: Quality improvement; RSI: Rapid sequence intubation; SED: Sedation only; VL: Video laryngoscope/laryngoscopy.

## Competing interests

The authors declare that they have no competing interests.

## Authors' contributions

JMM and JCS conceived the study and designed the data collection instrument. JMM managed the QI database with the assistance of LAG and GEC. JMM, SPW, JWB, LSS and JCS performed statistical analysis in the study. JMM, SPW, JWB, LSS, LAG, GEC and JCS contributed to the drafting of the manuscript. JMM takes responsibility for the paper as a whole. All authors read and approved the final manuscript.
